# Post-marketing safety re-evaluation of placental peptide injection in China: a large-scale multicenter real-world study

**DOI:** 10.3389/fphar.2025.1541005

**Published:** 2025-03-04

**Authors:** Liguang Duan, Feiyue An, Chen Tian, Wen Yan, Jie Chen, Hongyin Zhang, Xiaoguang Liu, Lingjiao Wang, Zhuo Zhang, Binliang Tong, Chunhua Zhou

**Affiliations:** ^1^ Pharmacy Department, The First Hospital of Hebei Medical University, Shijiazhuang, China; ^2^ Pharmaceutical Department, Kunming Third People’s Hospital, Kunming, China; ^3^ Pharmacy Department, Yunnan Cancer Hospital, Kunming, China; ^4^ Beijing Dehuijia Pharmaceutical Technology Co., Ltd., Beijing, China; ^5^ Department of Clinical Pharmacy, The First Hospital of Hebei Medical University, Shijiazhuang, China

**Keywords:** real-world study, placental peptide injection, post-marketing safety re-evaluation, adverse drug reactions, centralized hospital monitoring method

## Abstract

**Purpose:**

This study conducted a post-marketing safety re-evaluation of placental polypeptide injections in China to support updates to drug guidelines, pharmacovigilance efforts, and rational clinical use, facilitating its inclusion in essential drug lists and medical insurance coverage.

**Methods:**

A hospital-based centralized monitoring system tracked 3,000 patients receiving placental polypeptide injections across three medical institutions. Adverse drug reactions (ADRs) and adverse drug events (AEs) were systematically collected and analyzed.

**Results:**

The mean patient age was 49.65 years, with 96.47% being over 18 years of age. A single dose exceeding 4 mL was administered in 98.34% of the cases, with a median treatment duration of 7 days. Concomitant medication use was high (injectable, 98.43%; non-injectable, 75.43%). One case of vertigo was reported as an ADR in a patient aged >60 years who had melanoma.

**Conclusion:**

The ADR rate was 0.03%, confirming the favorable safety profile of placental polypeptide injection. These findings support its safe clinical use and can inform future regulatory and policy decisions.

## 1 Introduction

The human placenta, known as ZiheChe in Traditional Chinese Medicine (TCM), has been used medicinally for centuries because of its ability to nourish *qi*, blood, and essence, as well as its effects on the lung, heart, and kidney meridians (Wang et al., 2020). In modern clinical practice, placental polypeptide injection, a bioactive extract derived from the healthy human placenta, is widely used in China for the treatment of tumors, immune dysfunction, leukopenia, and other conditions.

Pharmacological studies have shown that placental polypeptide injections contain essential amino acids, active peptides, proteins, lipid fatty acids, nucleic acids, various growth factors and cytokines (Ye, 2002). Placental polypeptides are small functional peptides ranging from 3,000 to 5,000 Da in molecular weight, extracted, and purified using advanced biotechnological methods (Yuan et al., 2006; Tseng et al., 2012; Abd-Allah et al., 2015). These bioactive components play crucial roles in improving cellular immune function, scavenging free radicals, preventing peroxidation, and promoting the survival, proliferation, and differentiation of hematopoietic cells in the bone marrow. Placental polypeptides also enhance the activation and regulation of natural killer (NK) cells and T lymphocytes, thereby strengthening immune surveillance, cell-mediated immunity, and immune homeostasis (Fan and Fu, 2016). Furthermore, they mitigate bone marrow suppression caused by chemotherapy by promoting hematopoietic cell proliferation, reducing apoptosis, and improving hematopoietic function (Han, 2024). Owing to its demonstrated clinical efficacy, strong compatibility, affordability, and ability to alleviate economic burdens on patients, placental polypeptide injection has been widely applied in oncology, immunodeficiency disorders, leukopenia, and postoperative recovery from fractures (Yang, 2014). Previous studies, including a retrospective safety evaluation, reported no adverse reactions associated with its use, further supporting its clinical safety profile (Ou et al., 2020).

Despite these findings, there remains a critical need for comprehensive post-marketing safety evaluation of placental polypeptide injections. Traditional clinical trials are often limited by controlled conditions, small sample sizes, and restricted patient populations, which make it difficult to capture the full spectrum of drug safety in real-world settings. In contrast, real-world research (RWR) provides broader data sources, more diverse patient populations, and findings that closely reflect the actual clinical practice. The longitudinal nature of real-world studies allows for the collection of long-term outcome data, thereby improving the reliability of safety assessments. To bridge the gap in post-marketing safety data, this study employed a large-scale, multicenter approach to reassess the safety profile of placental polypeptide injection using real-world evidence (RWE). This evaluation will provide critical data to support updates to clinical guidelines, inform revisions to drug labeling, and strengthen evidence-based clinical applications. Additionally, it will contribute to safety monitoring, as placental polypeptide injection is considered for inclusion in essential drug lists, national medical insurance formularies, and centralized procurement policies, ensuring its safe and rational use in clinical practice.

## 2 Materials and methods

### 2.1 Research design and settings

This study employed a retrospective observational design that utilized centralized hospital-based safety monitoring. A large-scale multicenter surveillance cohort was established to assess the post-marketing safety of placental polypeptide injections. The study was conducted in accordance with the Declaration of Helsinki and Quality Management Standards for Pharmacovigilance to ensure compliance with ethical and regulatory requirements. Patients who received placental polypeptide injections were monitored across three tertiary hospitals: First Hospital of Hebei Medical University, Kunming Third People’s Hospital, and Yunnan Cancer Hospital.

### 2.2 Data collection and target variables

Data collection was performed using a standardized case report form (CRF), which included the following components: Patient General Information Record Table (Table A), Patient Treatment Drug Information Table (Table B), and Patient Adverse Event Record Table (Table C). The study monitored post-marketing data of placental polypeptide injections, recorded by trained pharmacovigilance researchers.

**FIGURE 1:**
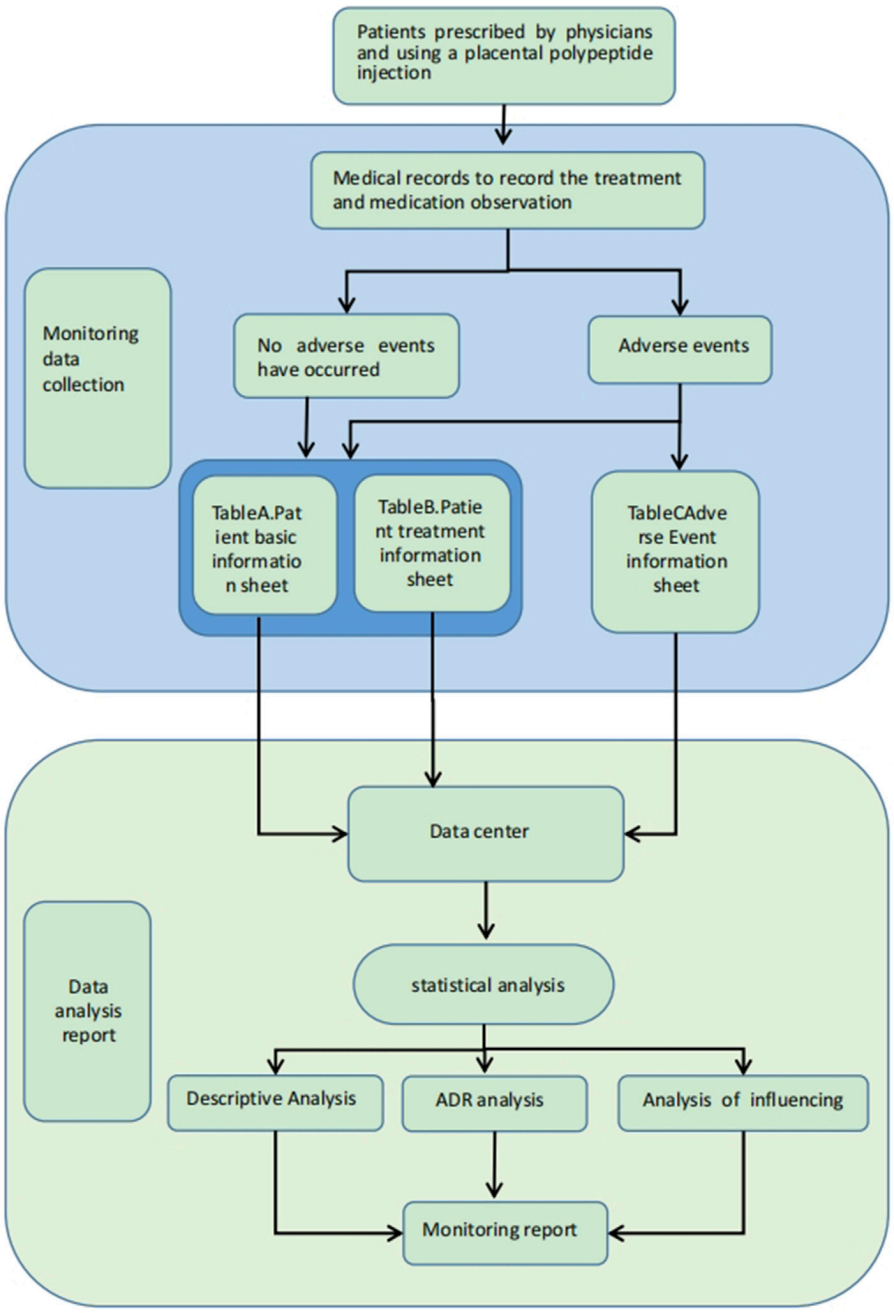
Flow chart of data collection.

The key variables included demographic characteristics, medical history, physical examination findings, vital signs, laboratory parameters, therapeutic regimens, and ADRs/AEs. A data collection flowchart is presented in Figure 1. All the collected data were submitted to a post-market surveillance database for further analysis.

The primary endpoints were the incidence and severity of ADRs and AEs. ADR was defined as any harmful response to a medicinal product occurring at normal therapeutic doses, and was not intended as part of the therapeutic effect. AEs were characterized as any untoward medical occurrence during treatment, regardless of their causal relationship with the investigational product. The severity of ADRs and AEs was evaluated using the Common Terminology Criteria for Adverse Events (CTCAE v5.0) version 5.0, established by the National Cancer Institute (NCI), in conjunction with the guidelines from the National Center for Adverse Drug Reactions’ ADR Reporting and Monitoring Workbook. Assessments of ADR/AE severity were conducted by experienced healthcare professionals at each participating institution.

### 2.3 Statistical analysis

The study utilized a single analysis set, the Primary Analysis Set (PAS), which included all the enrolled patients who received at least one dose of the study drug. All statistical analyses were performed using the SAS software (version 9.4). Continuous variables were summarized using mean, standard deviation, median, interquartile range (IQR), and minimum and maximum values. Categorical variables are presented as frequencies and percentages. Comparisons between groups were conducted using appropriate statistical methods based on the data type. For quantitative data, the paired t-test (for normally distributed data) or Wilcoxon signed-rank test (for non-normally distributed data) was used. For categorical data, the chi-square test or Fisher’s exact test was used (if any expected frequency was <4). For ordinal data, The Wilcoxon rank-sum test (without center effect correction) or the Cochran-Mantel-Haenszel (CMH) test (with center effect correction) was used. All statistical tests were two-sided with a significance threshold of p < 0.05, and results were reported with 95% confidence intervals (CIs).

## 3 Results

### 3.1 Baseline characteristics of the study population

The distribution of 3,000 patients across the three monitoring centers was as follows: 1,000 cases were monitored by Center 1 (The First Hospital of Hebei Medical University), 1,682 cases by Center 2 (Kunming Third People’s Hospital), and 318 cases by Center 3 (Yunnan Cancer Hospital). No cases of PAS were excluded, and all patients were successfully enrolled.

The baseline characteristics of the patients are summarized in Tables 1–4. The mean age of the patients was 49.65 ± 17.27 years. Regarding age distribution, 106 (3.53%) patients were aged 0–18 years, 948 (31.60%) were aged 18–45 years, 1,064 (35.47%) were aged 45–60 years, and 882 (29.40%) were aged ≥60 years. Of the total population, 1,723 (57.43%) were male, and 1,277 (42.57%) were female. In terms of ethnicity, 2,731 (91.03%) patients were of the Han ethnicity, while 269 (8.97%) belonged to other ethnic groups. Anthropometric data showed that the mean BMI, calculated from height and weight, was 22.32 ± 4.10 kg/m^2^. The mean body temperature of patients was 36.49°C ± 0.33°C, the mean pulse rate was 83.84 ± 13.84 beats/min, the mean respiration rate was 19.54 ± 2.03 breaths/min, and the mean systolic blood pressure was 119.27 ± 15.95 mmHg, with a mean diastolic blood pressure of 76.05 ± 10.65 mmHg. Regarding medical history, 1,849 (61.63%) patients had documented medical history, whereas 228 (7.60%) had a history of prior ADRs. Additionally, 1,849 (61.63%) patients presented with comorbidities. The detailed baseline demographic and clinical characteristics are provided in Tables 1–4.

**TABLE 1:** Demographic and anthropometric data.

Variable	Index	Statistical description
Age (years)	n (Missing)	3000 (0)
	Mean (SD)	49.65 (17.27)
	Median	51.27
	Q1, Q3	38.27, 62.95
	Min, Max	54, 93
Gender	Male n (%)	1723 (57.43)
	Female n (%)	1277 (42.57)
	Total (Missing)	3000 (0)
Ethnicity	Han Chinese n (%)	2731 (91.03)
	Other n (%)	269 (8.97)
	Total (Missing)	3000 (0)
Height (cm)	N (Missing)	3000 (153)
	Mean (SD)	163.36 (12.83)
	Median	165.00
	Q1, Q3	158.00, 170.00
	Min, Max	55.00, 187.00
Weight (kg)	N (Missing)	3000 (125)
	Mean (SD)	60.12 (13.46)
	Median	60.00
	Q1, Q3	52.00, 68.00
	Min, Max	5.00, 112.00
BMI (kg/m^2^)	N (Missing)	3000 (156)
	Mean (SD)	22.32 (4.10)
	Median	22.06
	Q1, Q3	19.62, 24.61
	Min, Max	8.84, 76.53

BMI: body mass index.

**TABLE 2:** Baseline vital signs before medication.

Variable	Index	Statistical description
Body Temperature (°C)	N (Missing)	3000 (1)
	Mean (SD)	36.49 (0.33)
	Median	36.50
	Q1, Q3	36.30, 36.60
	Min, Max	35.60, 40.40
Pulse (beats/min)	N (Missing)	3000 (1)
	Mean (SD)	83.84 (13.84)
	Median	81.00
	Q1, Q3	76.00, 90.00
	Min, Max	48.00, 188.00
Breathing (times/min)	N (Missing)	3000 (3)
	Mean (SD)	19.54 (2.03)
	Median	20.00
	Q1, Q3	18.00, 20.00
	Min, Max	12.00, 66.00
Systolic Blood Pressure (mmHg)	N (Missing)	3000 (1)
	Mean (SD)	119.27 (15.95)
	Median	119.00
	Q1, Q3	108.00, 129.00
	Min, Max	72.00, 212.00
Diastolic Blood Pressure (mmHg)	N (Missing)	3000 (1)
	Mean (SD)	76.05 (10.65)
	Median	75.00
	Q1, Q3	698.00, 82.00
	Min, Max	41.00, 125.00

**TABLE 3:** Medical and adverse drug reaction (ADR) history.

Variable	Index	Statistical description
Past Medical History	No n (%)	1151 (38.37)
	Yes n (%)	1849 (61.63)
	Total (Missing)	3000 (0)
Past ADR History	No n (%)	2772 (92.40)
	Yes n (%)	228 (7.60)
	Total (Missing)	3000 (0)
Comorbidities	No n (%)	1151 (38.37)
	Yes n (%)	1849 (61.63)
	Total (Missing)	3000 (0)

**TABLE 4:** Age group distribution.

Age (years)	n	%
0–18	106	3.53
18–45	948	31.60
45–60	1064	35.47
≥60	882	29.40

### 3.2 Clinical use of placental polypeptide injection

The real-world use of placental polypeptide injections is summarized in Tables 5–7. Among the 3,000 enrolled patients, the most common indications for treatment, each accounting for more than 1% of cases, are presented in Table 5. The most frequent indication was pulmonary tuberculosis (438 cases, 14.60%), followed by hepatitis B virus infection (229 cases, 7.63%) and hepatitis C virus infection (182 cases, 6.07%).

**TABLE 5:** Indications for placental polypeptide injection.

Disease indication	n	%
Pulmonary tuberculosis	438	14.60
Hepatitis B virus	229	7.63
Hepatitis C virus	182	6.07
Zoster	163	5.43
AIDS	153	5.10
Intestinal obstruction	132	4.40
Immune dysfunction	103	3.43
Pneumonia	96	3.20
Malignant tumor	82	2.73
Rectal cancer	72	2.40
Colon cancer	60	2.00
Hypoproteinemia	47	1.57
Gastric cancer	46	1.53
Decreased white blood cells	45	1.50
Lung cancer	43	1.43
Cellular immunodeficiency	43	1.43
Liver cancer	34	1.13
Ventricular septal defect	34	1.13

Most patients received a single dose of 8 mL (2,167 patients, 68.97%) or 4 mL (922 patients, 29.34%). The mean duration of medication use was 8.19 ± 5.79 days, with a median of 7 days. The mean total dose administered was 53.12 ± 41.36 mL, with a median total dose of 48 mL. The mean daily dose was 6.76 ± 2.22 mL, with a median of 8 mL (Table 6). A significant proportion of the patients received concomitant injectable medications, with 2,953 (98.43%) receiving at least one additional injectable drug. Furthermore, 2,263 patients (75.43%) received concomitant medications along with placental polypeptide injections (Table 7).

**TABLE 6:** Summary of drug administration and exposure.

Variable	Index	Statistical description
Single Dose Administration (mL)	N (Missing)	3143 (1)
	1, n (%)	22 (0.70)
	2, n (%)	30 (0.95)
	4, n (%)	922 (29.34)
	6, n (%)	1 (0.03)
	8, n (%)	2167 (68.97)
Average Medication Duration (days)	N (Missing)	3143 (0)
	Mean (SD)	8.19 (5.79)
	Median	7.00
	Q1, Q3	4.00, 11.00
	Min, Max	1.00, 6600
Total Dosage (mL)	N (Missing)	3143 (1)
	Mean (SD)	53.12 (41.36)
	Median	48.00
	Q1, Q3	24.00, 72.00
	Min, Max	100, 52800
Average Dose (mL/day)	N (Missing)	3143 (1)
	Mean (SD)	6.76 (2.22)
	Median	8.00
	Q1, Q3	4.00, 8.00
	Min, Max	1.00, 56.00

**TABLE 7:** Summary of concomitant medications.

Variable	Index	Statistical description
Use of Other Injectable Drugs	Yes n (%)	2953 (98.43)
	No n (%)	47 (1.57)
	Total (Missing)	3000 (0)
Use of Concomitant Medications	Yes n (%)	2263 (75.43)
	No n (%)	737 (24.57)
	Total (Missing)	3000 (0)

### 3.3 Adverse events and drug-related reactions

Among the 3,000 patients receiving placental polypeptide injections, 120 (4.0%) experienced at least one AE (Table 8). Of these, only one adverse event (0.03%) was related to the study drug, while no serious adverse events (SAEs) or treatment-related deaths were reported. This suggests that placental polypeptide injections have a favorable safety profile with a minimal risk of drug-induced reactions. Most AEs were classified as mild (106 cases, 3.53%), followed by moderate (16 cases, 0.53%). No severe adverse events were observed (Table 9). The most frequently reported adverse events were metabolic and nutritional disorders, neurological disorders, gastrointestinal disturbances, and hematologic abnormalities (Table 10). Common AEs included hyperuricemia (13 patients, 0.43%), constipation (14 patients, 0.47%), bone marrow suppression (7 patients, 0.23%), and dizziness (9 patients, 0.30%).

**TABLE 8:** Overview of adverse events.

Event type	Total number of events	Number of patients affected	Incidence rate (%)
Adverse Event	153	120	4.00
Drug-Related Adverse Reaction	1	1	0.03
Serious Adverse Events	0	0	0.00
Adverse Events Leading to Death	0	0	0.00

**TABLE 9:** Severity of adverse events.

Severity level	Total number of events	Number of patients affected	Incidence rate (%)
Mild	137	106	3.53
Moderate	16	16	0.53
Severe	0	0	0.00

**TABLE 10:** Classification of adverse events by system organ class (SOC).

System organ class (SOC)	Preferred term (PT)	Number of patients	Incidence rate (%)
Metabolic and Nutritional Diseases	Hypoproteinemia	4	0.13
	Electrolyte Imbalance	2	0.07
	Hyperuricemia	13	0.43
	Hyperhomocysteinemia	2	0.07
	Hyperlipidemia	1	0.03
	Decreased Appetite	1	0.03
Ear and Labyrinth Diseases	Vertigo	1	0.03
Diseases of the Liver and Gallbladder	Abnormal Liver Function	2	0.07
	Drug-Induced Liver Injury	2	0.07
Neurological Disorders	Dizziness	9	0.30
	Headache	3	0.10
	Tremor	1	0.03
Skin and Subcutaneous Tissue Diseases	Skin Reaction	2	0.07
Systemic Reactions	Fever	1	0.03
	Weakness	1	0.03
Gastrointestinal Disorders	Constipation	14	0.47
	Nausea	2	0.07
	Abdominal Discomfort	2	0.07
	Vomiting	2	0.07
Blood and Lymphatic System Disorders	Bone Marrow Suppression	7	0.23
	Neutropenia	1	0.03
	Coagulation Disorder	1	0.03
	Anemia	3	0.10

Only one adverse reaction (0.03%) was classified as related to placental polypeptide injection (Table 11). Reaction vertigo was coded under the MedDRA Preferred Term (PT) “Vertigo,” belonging to the System Organ Class (SOC) “Ear and Labyrinth Disorders.” According to the Council for International Organizations of Medical Sciences (CIOMS) severity criteria, this reaction is mild and does not require medical intervention. This case occurred in a patient with melanoma, an indication for placental polypeptide injection. Age-stratified analysis of adverse events (Table 12) showed the highest incidence rate (5.66%) in patients aged 0–18 years, followed by 4.64% in the 18–45 group, 4.42% in the 45–60 group, and 2.61% in those aged ≥60 years. Only one patient (0.11%) aged ≥60 years experienced an adverse event related to the study drug, while no study-related AEs were reported in the younger age groups.

**TABLE 11:** Summary of adverse drug reactions.

System organ class (SOC)	Preferred term (PT)	Number of patients	Incidence rate (%)
Ear and Labyrinth Diseases	Vertigo	1	0.03

**TABLE 12:** Summary of adverse events in different age groups.

Age (years)	Event type	Number of events	Number of patients affected	Incidence rate (%)
0–18	Adverse Event	6	6	5.66
	Drug-Related Adverse Events	0	0	0.00
18–45	Adverse Event	53	44	4.64
	Drug-Related Adverse Events	0	0	0.00
45–60	Adverse Event	58	47	4.42
	Drug-Related Adverse Events	0	0	0.00
≥60	Adverse Event	36	23	2.61
	Drug-Related Adverse Events	1	1	0.11

Overall, these findings indicate that placental polypeptide injections are well tolerated across all age groups, with a low incidence of ADRs and no reported SAEs.

### 3.4 Factors influencing adverse events

Linear regression analysis was conducted to examine the relationship between AE occurrence (dependent variable) and the following independent variables: age, sex, BMI, presence of comorbidities, concomitant medication use, and first-time use of placental polypeptide injection.

As shown in Table 13, the regression model yielded an *R* ^2^ value of 0.001, indicating that these independent variables explained only 0.1% of the variance in the AE incidence. The F-test for overall model significance showed F (6,2840) = 0.676, p = 0.669, which was greater than 0.05, confirming that the model was not statistically significant. None of the independent variables, age (β = −0.001, p > 0.05), sex (β = −0.001, p > 0.05), BMI (β = −0.000, p > 0.05), presence of comorbidities (β = 0.001, p > 0.05), concomitant medication use (β = 0, p > 0.05), or first-time use of placental polypeptide injections (β = 0, p > 0.05), exhibited a statistically significant association with the occurrence of AEs. Additionally, variance inflation factors (VIFs) for all predictors were close to one, indicating no multicollinearity concerns among the independent variables. The Durbin-Watson statistic (D-W=2.000) suggested no autocorrelation in the residuals, further supporting the model’s validity. Overall, these results indicate that age, sex, BMI, comorbidities, concomitant medication use, and first-time administration of placental polypeptide injections were not significant predictors of AE risk in this study population.

**TABLE 13:** Analysis of factors influencing adverse events.

Variable	Regression coefficient	95% CI	VIF	Tolerance
Coefficient	2.002**	1.994 ∼ 2.010	–	–
Intercept	−479.911	–	–	–
Age	−0.001 (−1.000)	−0.000 ∼ 0.000	1.04	0.961
Sex	−0.001 (−1.275)	−0.002 ∼ 0.000	1.01	0.99
BMI	−0.000 (−0.923)	−0.000 ∼ 0.000	1.029	0.972
Comorbidities	0.001 (−0.761)	−0.001 ∼ 0.002	1.006	0.994
Concomitant Medication	0 (−0.173)	−0.005 ∼ 0.006	1.002	0.998
First-Time Use of Placental Peptide Injection	0 (−0.245)	−0.003 ∼ 0.004	1.007	0.993
Sample Size	2847	–	–	–
*R* ^2^	0.001	–	–	–
Adjusted *R* ^2^	−0.001	–	–	–
F-statistic	F (6,2840) = 0.676, p = 0.669	–	–	–
D-W Statistic	2.000	–	–	–

*p < 0.05, **p < 0.01 (t-values in parentheses).

Note: Due to missing height or weight data in 153 patients, the valid sample size included in the analysis was 2,847.

This suggests that AEs to placental polypeptide injections occur independently of these common clinical variables, reinforcing the general safety of the drug across different patient profiles.

## 4 Discussion

The concept of RWR was first formally introduced by Kaplan et al., in 1993 through their study of ramipril in patients with hypertension (Kaplan et al., 1993). Real-world studies collect various types of data from diverse settings, including hospitals, communities, and households, and apply rigorous analytical frameworks to evaluate intervention outcomes in broader patient populations. The 21st Century Cures Act (21CCA) enacted by the U.S. In 2016, Congress (Bonamici, 2024) emphasized the importance of RWE in healthcare decision-making. Subsequently, the U.S. FDA issued its Guidance for Industry: Real-World Evidence Supporting Medical Device Management Decisions in 2017 (U.S.Food and Drug Administration, 2017), followed by the Real-World Evidence Program Framework in 2018 (FDA, 2018). Compared with traditional randomized controlled trials (RCTs), real-world studies incorporate broader patient populations, more diverse data sources, and extended follow-up periods, providing long-term clinical outcome observations (Fan, 2024). These attributes enhance the reliability of clinical trial design and regulatory decision making (Marzano et al., 2024).

In China, RWR has gained significant attention, particularly in the evaluation of TCM interventions (Zhong, 2009; Zengshi, 2009). Limitations of pre-marketing clinical trials of TCM, such as small sample sizes, restricted age groups, overly controlled medication regimens, and short treatment durations, necessitate post-marketing real-world evaluations (Feng and Xie, 2010). Real-world studies help to assess the effectiveness and safety of treatments across broader populations, considering real-world complexities (Sherman et al., 2016). The release of the Real-World Research Guide in 2018 (Wu et al., 2019) provided a structured framework for conducting high-quality RWR in China, leading to its widespread application (Can et al., 2018; Yang et al., 2011). Large-scale multicenter real-world studies have been instrumental in post-marketing safety evaluations of various drugs, including quinapril maleate (Guo et al., 2017), Longjing Tonglin capsules (Zhang et al., 2022), Shuxuening injection (Jin et al., 2023), Jiebiao Qingre Zhike decoction (Dezhong et al., 2023), ShumiTong capsules (Chunhe et al., 2021), and antiviral oral liquids (Hongjiao et al., 2022).

### 4.1 Key findings from this study

This large-scale, multicenter, real-world study investigated the postmarketing safety of placental polypeptide injections. The mean age of the patients was 49.65 years, with 96.47% aged >18 years. The mean body temperature was 36.49°C, with an average pulse rate of 83.84 beats per minute, respiratory rate of 19.54 breaths per minute, systolic blood pressure of 119.27 mmHg, and diastolic blood pressure of 76.05 mmHg. A history of prior disease was reported in 61.63% of the patients, 92.40% had a history of ADRs, and 61.63% had comorbid conditions.

Regarding medication use, most patients received doses exceeding 4 mL (98.34%), with an average treatment duration of 8.19 days and a mean total dose of 53.12 mL. Concomitant medication use was high, with 98.43% of patients receiving injectable co-medications and 75.43% receiving non-injectable treatments. These findings indicate that the clinical application of placental polypeptide injections adheres to the recommended dosage guidelines.

Despite its widespread use, only one adverse reaction (vertigo) has been reported. The affected patient exhibited symptom onset overlapping with placental polypeptide injection administration, and the symptoms resolved after discontinuation of the drug, suggesting a strong correlation between vertigo and treatment. The overall incidence of adverse reactions was 0.03%, classifying it as a rare adverse event based on CIOMS criteria.

### 4.2 Safety comparison with other immunomodulators

Placental polypeptide injections are commonly used as an immunomodulator and is often compared to thymosin injections, another widely used agent for malignancies, tuberculosis, pneumonia, and immune-related disorders. However, thymosin injections are associated with a significantly higher incidence of adverse reactions. From 2003 to 2011, 5,459 adverse reaction reports related to thymosin injections were documented in China (Chen et al., 2022). In early 2023, the National Medical Products Administration (NMPA) of China issued a black-box warning for thymosin injections, highlighting serious safety concerns. In contrast, placental polypeptide injections demonstrate a superior safety profile, with fewer reported adverse events and no severe reactions documented in this study.

### 4.3 Implications for drug safety monitoring and future research

This study highlights the importance of real-world hospital-based centralized monitoring for comprehensive, timely, and accurate drug safety evaluation. RWE offers an effective approach for post-marketing surveillance, ensuring better regulatory oversight and patient safety. Systematic collection of real-world data, interdisciplinary collaborations, and advanced analytical methodologies will facilitate the high-quality transformation of real-world data into actionable clinical evidence.

Post-marketing safety evaluations provide several benefits. They guide the rational and safe clinical use of medications, mitigate risks, and support drug risk-control strategies. Additionally, such evaluations contribute to enhancing drug labeling, updating safety warnings, and informing regulatory policies regarding pharmacovigilance, enforcement, and acceptance standards (Li et al., 2015).

### 4.4 Study limitations

This study has several limitations. First, this retrospective study may introduce the potential for selection bias, as patient inclusion depended on hospital-based documentation, which may not fully capture all adverse events. Additionally, underreporting of mild or transient adverse events is possible, as some symptoms may not have been recorded in the clinical notes. Second, the lack of a control group limited the ability to directly compare placental polypeptide injections with alternative therapies. Future studies incorporating matched controls or randomized comparisons would provide stronger evidence of relative safety and efficacy. Third, although this study included a diverse patient population, the findings may not be generalizable to all clinical settings. The study was conducted in three hospitals, and although they represented different regions, the results may not fully reflect safety profiles in primary care settings or smaller healthcare facilities. Finally, long-term safety data was limited. Although real-world studies enable a longer follow-up than traditional trials, the study duration was insufficient to evaluate delayed or chronic adverse effects. Further long-term observational studies or registry-based surveillance would help to assess potential late-onset safety concerns.

## 5 Conclusion

This large-scale, multicenter, real-world study demonstrated that placental polypeptide injections exhibit a good clinical safety profile, with an adverse reaction rate of only 0.03%. Most adverse events were mild, and gastrointestinal disorders were the most frequently observed. These findings provide critical pharmacovigilance evidence to support clinical guideline updates, regulatory decisions, and the inclusion of placental polypeptide injections in national essential medicine lists, insurance schemes, and procurement policies, ensuring its safe and rational clinical use.

## Data Availability

The original contributions presented in the study are included in the article/supplementary material, further inquiries can be directed to the corresponding authors.
